# Lower Performance in Orientation to Time and Place Associates with Greater Risk of Cardiovascular Events and Mortality in the Oldest Old: Leiden 85-Plus Study

**DOI:** 10.3389/fnagi.2017.00307

**Published:** 2017-09-27

**Authors:** Somayeh Rostamian, Mark A. van Buchem, J. Wouter Jukema, Jacobijn Gussekloo, Rosalinde K. E. Poortvliet, Anton J. M. de Cren, Behnam Sabayan

**Affiliations:** ^1^Department of Radiology, Leiden University Medical Center, Leiden, Netherlands; ^2^Department of Gerontology and Geriatrics, Leiden University Medical Center, Leiden, Netherlands; ^3^Department of Cardiology, Leiden University Medical Center, Leiden, Netherlands; ^4^Department of Public Health and Primary Care, Leiden University Medical Center, Leiden, Netherlands; ^5^Feinberg School of Medicine, Northwestern University, Chicago, IL, United States

**Keywords:** orientation to time, orientation to place, myocardial infarction, stroke, mortality, oldest old

## Abstract

**Background:** Impairment in orientation to time and place is commonly observed in community-dwelling older individuals. Nevertheless, the clinical significance of this has been not fully explored. In this study, we investigated the link between performance in orientation domains and future risk of cardiovascular events and mortality in a non-hospital setting of the oldest old adults.

**Methods:** We included 528 subjects free of myocardial infarction (Group A), 477 individuals free of stroke/transient ischemic attack (Group B), and 432 subjects free of both myocardial infarction and stroke/transient ischemic attack (Group C) at baseline from the population-based Leiden 85-plus cohort study. Participants were asked to answer five questions related to orientation to time and five questions related to orientation to place. 5-year risks of first-time fatal and non-fatal myocardial infarction, fatal and non-fatal stroke, as well as cardiovascular and non-cardiovascular mortality, were estimated using the multivariate Cox regression analysis.

**Results:** In the multivariable analyses, adjusted for sociodemographic characteristics and cardiovascular risk factors, each point lower performance in “orientation to time” was significantly associated with higher risk of first-time myocardial infarction (hazard ratio [HR] 1.35, 95% confidence interval [CI] 1.09–1.67, *P* = 0.007), first-time stroke (HR 1.35, 95% CI 1.12–1.64, *P* = 0.002), cardiovascular mortality (HR 1.28, 95% CI 1.06–1.54, *P* = 0.009) and non-cardiovascular mortality (HR 1.37, 95% CI 1.20–1.56, *P* < 0.001). Similarly, each point lower performance in “orientation to place” was significantly associated with higher risk of first-time myocardial infarction (HR 1.67, 95% CI 1.25–2.22, *P* = 0.001), first-time stroke (HR 1.39, 95% CI 1.05–1.82, *P* = 0.016), cardiovascular mortality (HR 1.35, 95% CI 1.00–1.82, *P* = 0.054) and non-cardiovascular mortality (HR 1.45, 95% CI 1.20–1.77, *P* < 0.001).

**Conclusions:** Lower performance in orientation to time and place in advanced age is independently related to higher risk of myocardial infarction, stroke and mortality. Impaired orientation might be an early sign of covert vascular injuries, putting subjects at greater risk of cardiovascular events and mortality.

## Introduction

Current evidence indicates that impaired cognitive function is associated with cardiovascular events and mortality (O'Donnell et al., 2012). An increasing body of evidence from neuroimaging studies has shown that older subjects with cognitive dysfunction have a greater load of subclinical brain vascular abnormalities including white matter lesions, silent infarcts and microbleeds. Such brain vascular pathologies can predispose individuals to future cerebrovascular events and mortality (Schmidt et al., 2005; Bokura et al., 2006). Hence, cognitive impairment might be an early manifestation of clinically unrecognized cerebral and systemic vascular pathologies which signals future risk of cardiovascular events (Rostamian et al., 2014, 2015).

Most of the studies that investigated the association of cognitive impairment with future risk of cardiovascular events were performed in middle-aged and young old people (de Galan et al., 2009; Wiberg et al., 2010; O'Donnell et al., 2012). Thus, it is not clear whether these findings can be extrapolated to the rapidly growing population of very old subjects over 85 years of age, so called the oldest old (Weverling-Rijnsburger et al., 2003; Oates et al., 2007). Moreover, due to the long administration time and complexity, many of common cognitive tests are challenging and have proved to lead to fatigue, frustration and uncooperativeness in the oldest old (Ravdin et al., 2004; Whittle et al., 2007). Although previous research assessed the link between global cognitive function and mortality (Park et al., 2013; Batty et al., 2016), there is a limited evidence available on the association between performance in orientation domains and future risk of cardiovascular and non-cardiovascular mortality and cardiovascular events, especially in the oldest old subjects.

Orientation to time and place are two commonly used cognitive assessments that do not require expertise and can be applied in various settings. Despite the fact that impairment in orientation domains is common among older subjects, the clinical significance of this has not been fully explored. Accordingly, we aimed to investigate on the risk of cardiovascular events and mortality dependent on performance in orientation domains. The purpose of this study is to determine the link between performance in orientation to time and orientation to place with future risk of cardiovascular events and mortality in a non-hospital setting of the population-based cohort of the oldest old individuals free of previous cardiovascular events.

## Methods

### Participants and procedures

Leiden 85-plus Study is a prospective population-based study of inhabitants of Leiden district, the Netherlands. Between September 1st, 1997; and September 1st, 1999, 705 inhabitants of Leiden reached the age of 85 were eligible to participate. There were no selection criteria based on health or demographic characteristics. The medical ethical committee of the Leiden University Medical Center approved the study. Oral and written information about the study were provided, and informed consent was obtained from all subjects. 599 individuals (response rate of 85%) participated in the study. Within a month after their 85th birthday, a physician or a research nurse contacted them by phone to request their participation. If subjects agreed to participate, they were visited at their place of residence. For this study, we excluded three subjects with uncompleted cognitive tests at baseline. Furthermore, based on outcome variable, we excluded subjects with the previous clinical history of myocardial infarction (Group A), clinical stroke and/or transient ischemic attack (TIA) (Group B), and from history of both myocardial infarction and stroke/TIA events (Group C) at baseline. Information obtained from general practitioners or nursing home physicians. In total, data of 528 subjects free of clinical myocardial infarction (Group A) and 477 subjects free of clinical stroke/TIA (Group B) at baseline were analyzed to evaluate the risk of future myocardial infarction and stroke respectively. Additionally, 432 subjects without a history of both myocardial infarction and stroke/TIA events (Group C) at baseline were examined for cumulative risk of cardiovascular events. Moreover, subjects in Group C were further tested for 5-year risks of cardiovascular, non-cardiovascular and all-cause mortality.

### Orientation to time and place

Data on orientation domains were extracted from standard administrated Mini-Mental State Examination (MMSE) at the time of entry to the study. To evaluate orientation domains, each participant was asked to answer five questions related to orientation to time (date, day of week, month, season, and year) and five questions related to orientation to place (country, province, city, street, and house number) and were received scores ranged from zero to five. Lower scores in each test indicate greater impairment in orientation domains. Risks of cardiovascular events and mortality were assessed continuously per each point lower scores in orientation to time and orientation to place.

### Outcomes

First fatal and non-fatal myocardial infarction, stroke, and cumulative cardiovascular events were considered as the main outcomes. Moreover, 5-year cardiovascular, non-cardiovascular and all-cause mortality were assessed. Information about health status and events were recorded with general practitioners. The occurrence of clinically recognized myocardial infarction and stroke during 5 years of follow-up was assessed by annually interviewing general practitioners and were recorded according to the International Classification of Disease and Related Disorders, Tenth revision. Death caused by myocardial infarction was classified as ICD-10 codes I21-I22 (Schramm et al., 2011) and death caused by stroke was classified as ICD-10 codes I60–I69 (Kokotailo and Hill, 2005).

### Covariates

Sociodemographic data and medical history of all participants were recorded at baseline. Education was dichotomized as either low education and unskilled profession or high education and skilled profession. Body mass index and blood pressure were measured by trained personnel. Total cholesterol level was analyzed on fully automated computerized analysers (Hitachi 747 and 911; Hitachi, Ltd., Tokyo, Japan). All participants were interviewed for the current and past history of smoking. Diabetes mellitus was considered present when included in the records of the primary care physician when non-fasting glucose concentrations were more than 11.0 millimoles per litter, or when a participant was using anti-diabetic medication according to their pharmacy records. Hypertension was defined as the history of hypertension, or use of antihypertensive medication at baseline. History of cardiovascular diseases, including atrial fibrillation, myocardial infarction and stroke/TIA was obtained from general practitioners or nursing home physicians at baseline.

### Statistical analyses

Baseline characteristics of subjects reported as mean with standard deviation for continuous variables and frequency with the percentage for categorical variables. We calculated incidence rates by dividing the number of events by person-years at risk during the follow-up period. The incident myocardial infarction and incident stroke and mortality in relation to orientation to time and place were calculated with Cox regression and reported by hazard ratio (HR) with 95% confidence interval (CI) in three models. Firstly, we calculated hazard ratios for the association between orientation domains and outcomes (crude model). In the second model, analyses were adjusted for sex and education (adjusted model 1). Finally, analyses were further adjusted for current smoking, body mass index, total cholesterol, systolic blood pressure, history of hypertension, history of diabetes mellitus, history of atrial fibrillation and history of previous stroke/TIA events in subjects free of myocardial infarction or history of previous myocardial infarction events in subjects free of stroke/TIA (adjusted model 2). Moreover, we tested whether the risk of cardiovascular events was independent of the clinical cut-off points of the MMSE test in impaired (MMSE < 24) vs. preserved (MMSE ≥ 24) level of cognitive function. P for interaction was calculated by adding an interaction term produced by multiplying scores of orientation to time and place and stratified variable. All analyses were conducted using SPSS statistical software (SPSS for Windows, version 20, SPSS Inc., Chicago, IL).

## Results

Table 1 summarizes baseline characteristics of the participants in three groups, free of myocardial infarction (Group A), free of stroke/TIA (Group B), and free of both cardiovascular events (Group C). During 5 years of follow-up, 31 out of 528 subjects (5.9%) in Group A experienced their first myocardial infarction event (incidence rate: 15.8; 95% CI 11.0–22.4) and 50 out of 477 subjects (10.5%) in Group B experienced their first stroke event (incidence rate: 27.4; 95% CI 20.8–36.0).

**Table 1:** Baseline characteristics of participants.

**Characteristics**	**Group A (*n* = 528)**	**Group B (*n* = 477)**	**Group C (*n* = 432)**
Male, *n* (%)	172 (32.6)	160 (33.5)	137 (31.7)
Low education or unskilled profession, *n* (%)	334 (63.3)	308 (64.6)	272 (63.0)
Current smoking, *n* (%)	84 (15.9)	78 (16.4)	70 (16.2)
Body mass index, kg/m^2^, mean (SD)	27.3 (4.6)	27.3(4.54)	27.4 (4.7)
Total cholesterol, mmol/L, mean (SD)	5.7 (1.1)	5.8 (1.1)	5.7 (1.1)
Systolic blood pressure, mmHg, mean (SD)	156.0 (18.9)	155.61 (18.3)	156.5 (18.3)
History of hypertension, *n* (%)	198 (37.5)	173 (36.3)	152 (35.2)
History of diabetes mellitus, *n* (%)	80 (15.2)	72 (15.1)	63 (14.6)
History of atrial fibrillation, *n* (%)	50 (9.5)	40 (8.4)	35 (8.1)
History of stroke/TIA, *n* (%)	93 (17.7)^*^	–	–
History of myocardial infarction, *n* (%)	–	43 (9.0)^*^	–

*Group A comprises subjects free of myocardial infarction; Group B includes individuals free of stroke/transient ischemic attack; and Group C contains subjects free of both myocardial infarction and stroke/transient ischemic attack at baseline of Leiden 85-plus cohort study. MI, Myocardial Infarction; TIA, Transient Ischemic Attack; CV, Cardiovascular; n, number; kg/m^2^, kilogram per meter squared; SD, Standard Deviation; mmol/L, millimoles per litter; mmHg, millimeters of mercury*.

* *Three subjects had missing data on history of myocardial infarction and two subjects had missing data on history of stroke/TIA at baseline*.

Table 2 shows the risk of first fatal and non-fatal myocardial infarction, stroke, and cumulative cardiovascular events in association with orientation to time and place in the crude, minimally and fully adjusted models. In the fully adjusted model, one point lower score of orientation to time was associated with 1.35-fold higher risk (95% CI 1.09−1.67; *P* = 0.007) of myocardial infarction in Group A and 1.35-fold higher risk (95% CI 1.12−1.64; *P* = 0.002) of stroke in Group B. Similarly, one point lower score in orientation to place was associated with 1.67-fold higher risk (95% CI 1.25−2.22; *P* = 0.001) of myocardial infarction in Group A and about 1.39-fold higher risk (95% CI 1.05−1.82; *P* = 0.016) of stroke in Group B. Overall, each point lower score in orientation to time in Group C was associated with about 1.39-fold (95% CI 1.18−1.64; *P* < 0.001) and each point of decline in orientation to place was associated with around 1.52-fold (95% CI 1.20−1.92; *P* = 0.001) higher chance of cardiovascular events.

**Table 2:** 5-year risks of cardiovascular events in relation to scores on orientation to time and place at age 85 years.

	**Cardiovascular events**					
	**Incident myocardial infarction in Group A (*****n*** = **528)**		**Incident stroke in Group B (*****n*** = **477)**		**Incident cumulative cardiovascular events in Group C (*****n*** = **432)**	
	**HR (95% CI)**	***P*-value**	**HR (95% CI)**	***P*-value**	**HR (95% CI)**	***P*-value**
**ORIENTATION TO TIME**						
Crude	1.25 (1.05–1.49)	0.013	1.32 (1.10–1.56)	0.002	1.29 (1.11–1.50)	0.001
Model 1	1.32 (1.09–1.61)	0.005	1.30 (1.09–1.54)	0.004	1.33 (1.14–1.55)	<0.001
Model 2	1.35 (1.09–1.67)	0.007	1.35 (1.12–1.64)	0.002	1.39 (1.18–1.64)	<0.001
**ORIENTATION TO PLACE**						
Crude	1.35 (1.08–1.69)	0.008	1.33 (1.06–1.69)	0.013	1.32 (1.07–1.62)	0.009
Model 1	1.52 (1.18–1.92)	0.001	1.33 (1.04–1.69)	0.023	1.39 (1.13–1.73)	0.002
Model 2	1.67 (1.25–2.22)	0.001	1.39 (1.05–1.82)	0.016	1.52 (1.20–1.92)	0.001

*Group A comprises subjects free of myocardial infarction; Group B includes individuals free of stroke/transient ischemic attack; and Group C contains subjects free of both myocardial infarction and stroke/transient ischemic attack at baseline of Leiden 85-plus cohort study. n, number; HR, Hazard Ratio; CI, Confidence Interval. Model 1, Adjusted for sex and education. Model 2, Adjusted for sex, education, current smoking, body mass index, total cholesterol, systolic blood pressure, history of hypertension, history of diabetes mellitus, history of atrial fibrillation and history of stroke/transient ischemic attack in subjects free of myocardial infarction or history of coronary events in subjects free of stroke/transient ischemic attack. Hazard ratios were calculated in association with each score lower performance in orientation scores and risk of cardiovascular events*.

Table 3 demonstrates the risk of mortality in five years in relation to orientation scores which was evaluated in Group C (individuals free of cardiovascular events at baseline). Mean age with the standard deviation of survival during the follow-up time was 3.69 (1.65) years for cardiovascular mortality and 3.98 (1.48) years for non-cardiovascular mortality. Our findings showed that poor performance in orientation to time was associated with the increased risk of 5-year cardiovascular (HR 1.28, 95% CI 1.06–1.54; *P* = 0.009), non-cardiovascular (HR 1.37, 95% CI 1.20–1.56; *P* < 0.001), and all-cause (HR 1.41, 95% CI 1.27–1.56; *P* < 0.001) mortality. Similar findings were observed for the association between performance in orientation to place with 5-year cardiovascular (HR 1.35, 95% CI 1.00–1.82; *P* = 0.054), non-cardiovascular (HR 1.45, 95% CI 1.20–1.77; *P* < 0.001), and all-cause (HR 1.56, 95% CI 1.34–1.80; *P* < 0.001) mortality.

**Table 3:** 5-year risks of mortality in relation to scores on orientation to time and place in Group C (free of cardiovascular events) at age 85 years.

	**Mortality**					
	**Cardiovascular (*****n*** = **432)**		**Non-cardiovascular (*****n*** = **432)**		**All-cause (*****n*** = **432)**	
	**HR (95% CI)**	***P*-value**	**HR (95% CI)**	***P*-value**	**HR (95% CI)**	***P*-value**
**ORIENTATION TO TIME**						
Crude	1.22 (1.03–1.45)	0.023	1.43 (1.29–1.58)	<0.001	1.39 (1.28–1.51)	<0.001
Model 1	1.23 (1.03–1.47)	0.019	1.43 (1.29–1.60)	<0.001	1.41 (1.29–1.55)	<0.001
Model 2	1.28 (1.06–1.54)	0.009	1.37 (1.20–1.56)	<0.001	1.41 (1.27–1.56)	<0.001
**ORIENTATION TO PLACE**						
Crude	1.18 (0.88–1.56)	0.264	1.52 (1.34–1.74)	<0.001	1.46 (1.31–1.63)	<0.001
Model 1	1.20 (0.89–1.61)	0.219	1.57 (1.35–1.82)	<0.001	1.55 (1.37–1.75)	<0.001
Model 2	1.35 (1.00–1.82)	0.054	1.45 (1.20–1.77)	<0.001	1.56 (1.34–1.80)	<0.001

*n, number; HR, Hazard Ratio; CI, Confidence Interval. Model 1, Adjusted for sex and education. Model 2, Adjusted for sex, education, current smoking, body mass index, total cholesterol, systolic blood pressure, history of hypertension, history of diabetes mellitus, history of atrial fibrillation and history of stroke/transient ischemic attack in subjects free of myocardial infarction or history of coronary events in subjects free of stroke/transient ischemic attack. Hazard ratios were calculated in association with each score lower performance in orientation scores and risk of cardiovascular events*.

**Figure 1:**
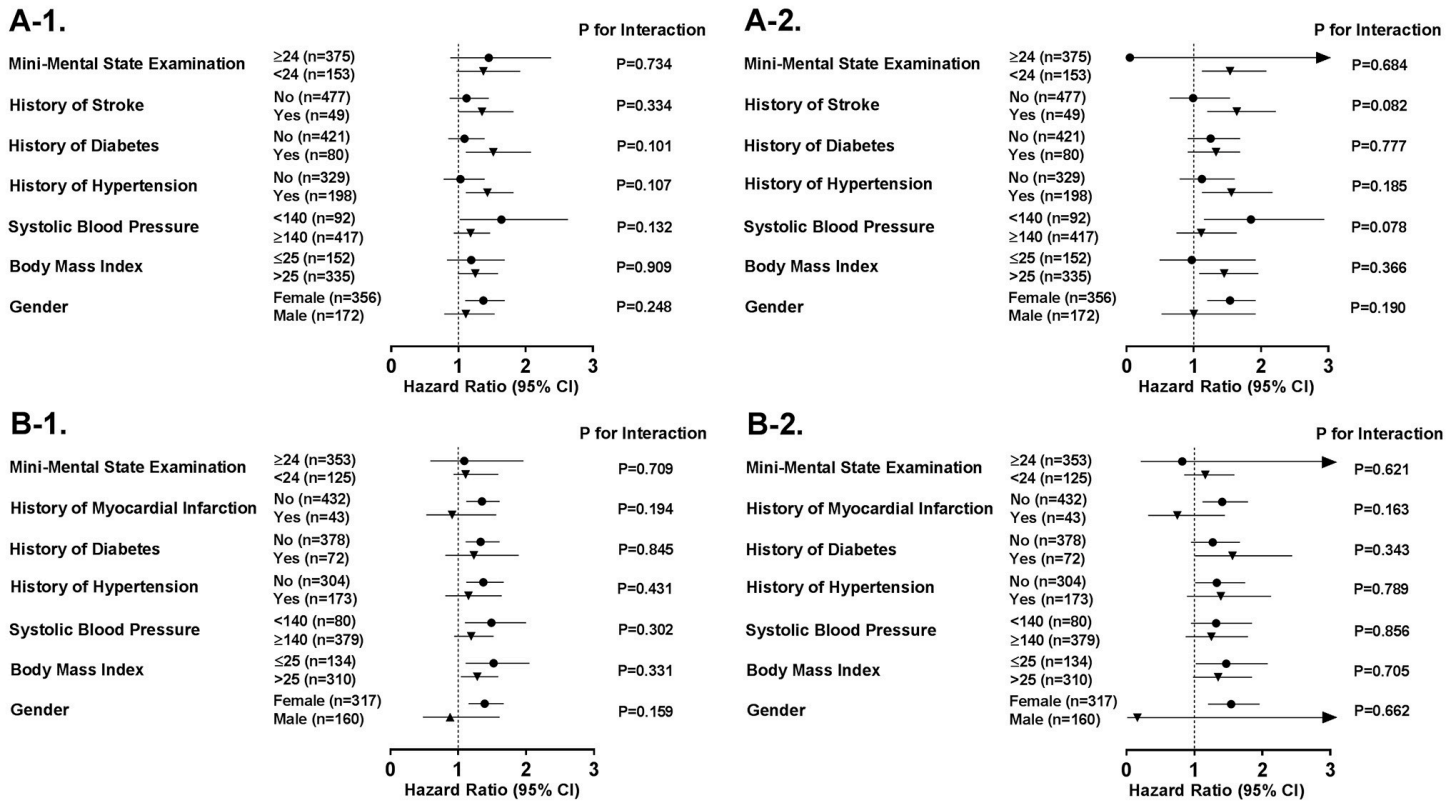
Subgroup analyses on the association between performance in orientation tests and risk of cardiovascular events. Sensitivity analyses show risk of **(A-1)** myocardial infarction in association to performance in orientation to time, **(A-2)** myocardial infarction in association to performance in orientation to place, **(B-1)** stroke in association to performance in orientation to time, and **(B-2)** stroke in association to performance in orientation to place, in different subgroups of cardiovascular risk factors and diseases. CI, Confidence Interval; n, number; TIA, Transient Ischemic Attack.

Figure 1 demonstrates a series of stratified analyses in group A and B in which we showed that the association of orientation to time and place with the incident myocardial infarction and incident stroke was comparable in various strata in different subgroups of participants with and without risk factors and comorbidities. Furthermore, we demonstrated that this association was similar in subjects with clinical cut-off points of impaired and preserved cognitive performance of MMSE score (MMSE < 24 vs. MMSE ≥ 24). These analyses showed the consistency of our findings in various subgroups (all *P* for interaction <0.05).

## Discussion

In this population-based study of the oldest old individuals, we present that lower performance in orientation to time and place, independent of socio-demographic and conventional cardiovascular risk factors, is associated with greater risk of incident myocardial infarction in Group A (free of myocardial infarction at baseline), stroke in Group B (free of stroke/TIA at baseline), and cumulative cardiovascular events in Group C (free of both myocardial infarction and Stroke/TIA at baseline). Moreover, when we examined mortality due to cardiovascular or non-cardiovascular causes in Group C, it is increased in relation to poorer performance in orientation to time and place.

The oldest old population is the fast growing segment of the western population (Robine and Paccaud, 2005). Since, the oldest old individuals carry a great burden of disease and disability, substantial concerns have been expressed regarding the future ability of the health care system to provide needed care (Wetle, 2008). The risk of future cardiovascular diseases, as the main cause of morbidity and mortality in almost all industrialized countries and in many developing countries, was assessed in different ways and especially in middle age and young elderly, to build-up prevention strategies (Perk et al., 2012). Although detecting of the oldest old cases at the higher risk of cardiovascular events and mortality is desirable for early interventions, treatment and planning of healthcare, yet, several studies failed to show the association between conventional cardiovascular risk factors and risk of future cardiovascular events and mortality in late life (Odden et al., 2014).

Several studies showed that orientation to time and place are among the first domains of cognitive function that get affected in elderly individuals and among the earliest domains to be lost in dementia (Fillenbaum et al., 1994; Guerrero-Berroa et al., 2009). As a result, evaluation of orientation domains can be applied to distinguish between different stages of cognitive dysfunctioning and dementia (Schultz-Larsen et al., 2007). For optimal functioning of the brain and the maintenance of cognitive functions during aging, neural plasticity and integrity of the brain are essential (Burke and Barnes, 2006). Ageing-related degeneration of structure and function of the cerebral vasculature system may disturb local perfusion and cerebral integrity (Kalaria, 1996). Impaired orientation might be a sign of disruption of neural plasticity and cerebral integrity as well as an early manifestation of covert vascular pathologies, putting subjects at the greater risk of cardiovascular events and mortality.

Our findings on the association between poor performance in orientation to time and place with higher incident cardiovascular events and mortality are in line with previous reports, demonstrating that impairment in orientation to time and place was associated with higher incident cardiovascular events and lower survival among elderly (O'Donnell et al., 2012; Iwasa et al., 2013; Park et al., 2013; Takata et al., 2014). Therefore, evaluation of global as well as domains-specific cognitive function such as orientation can be considered as a risk assessment for cardiovascular events and mortality in elderly subjects. Moreover, administration of complex cognitive tests is challenging in very old subjects and seniors are not able to stand complex time-consuming tests (Ravdin et al., 2004). Orientation tests are simple measures that can be applied by healthcare professionals with the various level of skills. Unlike other complex neuropsychological tests, applying orientation test does not require specific training. With further investigations it can be stated that evaluation of orientation domains, can be counted as a potential easily accessible tool, alone or in combination with established predictive methods to detect oldest old adults at the higher risk of cardiovascular events and mortality.

Our study has strengths and limitations. As strength, this study included relatively large population of the oldest old individuals with long follow-up time and availability of extensive data on sociodemographic, cardiovascular factors and mortality causes. The first possible limitation is the lack of neuroimaging data to identify subjects with covert brain vascular abnormalities at baseline. As a second possible limitation, this study was conducted in a sample of very old Dutch population, and thus, the results should be confirmed in other geographic, racial or ethnic subjects. The third limitation of the study is the lack of evidence of physical activity and mental health of participants which could potentially influence the results.

In conclusion, our findings suggest that lower performance in orientation to time and place, in the oldest old individuals, is associated with incident myocardial infarction, incident stroke and mortality. This evidence might suggest that applying simple, fast and practical tests like orientation to time and place can be further considered for enhancing prediction models for identifying the oldest old subjects who are at the increased risk of cardiovascular events and cerebrovascular accidents. Since previous studies that evaluated the association between cognitive function and future risk of cardiovascular events and mortality were performed mostly in the white middle aged and young elderly populations, the generalizability of those findings is limited. To improve the external validity, different age groups of elderly with diverse ethnic background and lifestyle are needed to be included in the future studies.

## Author contributions

Study concept and design: BS, SR, and AdC. Data analysis: SR. Data acquisition and interpretation, critical revising of the manuscript, and agreement to be accountable for all aspects of the work: SR, MvB, JJ, JG, RP, AdC, and BS. Drafting of the manuscript: SR and BS. Final approval of the version to be published: SR, MvB, JJ, JG, RP, and BS.

### Conflict of interest statement

The authors declare that the research was conducted in the absence of any commercial or financial relationships that could be construed as a potential conflict of interest.
